# Porous Silicon Structures as Optical Gas Sensors

**DOI:** 10.3390/s150819968

**Published:** 2015-08-14

**Authors:** Igor A. Levitsky

**Affiliations:** Emitech, Inc., Fall River, MA 02720, USA; E-Mail: ilevitsky@emitechinc.com; Tel.: +1-508-324-0758

**Keywords:** porous silicon, gas sensors, optical, sensor arrays, multiparametric

## Abstract

We present a short review of recent progress in the field of optical gas sensors based on porous silicon (PSi) and PSi composites, which are separate from PSi optochemical and biological sensors for a liquid medium. Different periodical and nonperiodical PSi photonic structures (bares, modified by functional groups or infiltrated with sensory polymers) are described for gas sensing with an emphasis on the device specificity, sensitivity and stability to the environment. Special attention is paid to multiparametric sensing and sensor array platforms as effective trends for the improvement of analyte classification and quantification. Mechanisms of gas physical and chemical sorption inside PSi mesopores and pores of PSi functional composites are discussed.

## 1. Introduction

It would not be an overestimation to say that porous silicon (PSi) optical sensors take a unique and important position in the field of chemo- and bio- sensing. The attractive features of PSi structures are not only the high surface area but also a controllable fabrication of porous layers via electrochemical anodization, allowing for the creation of a diversity of pores of different morphology and size, ranging from a few nanometers to microns. Another critical advancement is the possibility of preparing complex multilayer structures (Bragg reflectors, Rugate filters, microcavities, nonperiodic 1D structures, 2D photonic crystals, *etc.*) through variation of the current density, electrolyte formulation, temperature, etching time, and resistivity of crystalline Si [1]. Such structures cannot principally be achieved in other porous materials (e.g., aluminum oxides, zeolites), making PSi an ideal candidate for sensing applications. In addition, PSi can be easily functionalized by either using methods of surface chemistry or being infiltrated with other materials providing the specificity to target molecules. At a sufficient doping level, the porous silicon can be highly conductive and thus responsive to analytes in the electrical domain in parallel with optical signals, providing a basis for multiparametric sensing. Finally, PSi fabrication is relatively simple, inexpensive, and fully compatible with a microprocessing technique that allows for the integration of PSi sensors with other silicon modules in one microchip.

The detection of chemical and biological species using optical transduction in PSi structures were extensively investigated for the past twenty five years since the first reports of Sailor’s group about changing optical properties of the PSi monolayer upon exposure to several organic vapors [2,3]. Later, the detection of volatile organic compounds (VOCs), inorganic gases, explosives, organophosphates, DNA, proteins, and other biomolecules in aqueous medium have been reported. Several recent reviews described the progress and challenges in the field of PSi chemical and biological sensors [4,5,6]. All of them cover the entire area of PSi sensing (chemo-, bio-, vapors, liquids, electrical, and optical readouts) without a specific selection of transduction methods, and a phase of analyte molecules (liquid/vapor). Therefore, we believe that this short review, focusing only on optical methods for vapors detection, will be relevant for the systematization of different sensing approaches and appropriated PSi structures. PSi optical biosensing and chemosensing in a liquid medium (e.g., metal ions, toxins dissolved in water) and PSi electrical devices (except multiparametric detection involving optical signals) are beyond the scope of this review.

**Figure 1:**
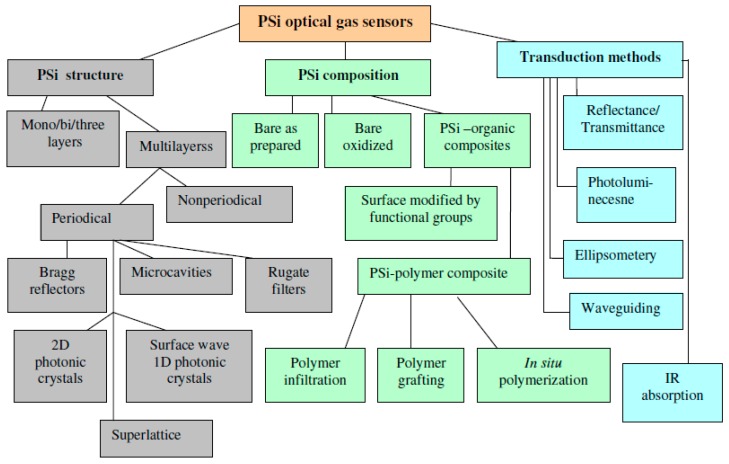
Major components of PSi optical gas sensors.

During recent decades, gas sensing has attracted great attention in academia and industry due to its widespread applications in environmental monitoring, homeland security, industrial emission control, noninvasive breath diagnostics, and food quality control [7]. Optical gas sensors have one critical advancement over gas detectors employing electrical readout (resistive, capacitive, surface acoustic wave gravimetric, *etc.*): the lack of necessity for metal contacts and wire coupling. They can be deployed in inaccessible locations and harsh environments using standoff interrogation followed by signal processing. However, similar to other sensing devices, optical gas detectors face several key challenges, which include: sensitivity, specificity, interferant discrimination, response and recovery time, background drift, and aging. Fortunately, for the porous Si, some of the above challenges can be solved due to the incredible diversity of PSi structures, material morphology, surface chemistry, and optical transduction mechanisms. Figure 1 depicts the major components of PSi optical gas sensors which can be systemized in three large groups: PSi structure, PSi composition, and optical transduction methods. Note that the diagram in Figure 1 is related to the single (or point) sensors, and the PSi sensor arrays will be considered in Section 5. 

There is a vast and growing amount of studies in this area, aiming to improve sensors’ performance via sophisticated surface functionalization, novel architectures, multiparametric readout, and sensor array platform, along with pattern recognition algorithms and intelligent signal processing. This review mainly focuses on recent advances in the field of PSi optical gas detectors in order to systemize and highlight the most promising trends and strategies toward the system improvement for gas sensing applications. After a short overview of early works related to optical transduction mechanisms and PSi based structures, the key topics in the current and near future research will be presented and discussed.

## 2. Early Studies and Mechanisms of Optical Gas Sensing

In the early 90s, Sailor’s group reported photoluminesenece (PL) quenching of PSi monolayer upon exposure to benzene, ethanol, several other VOCs, and water saturated vapors [2,3]. The quenching degree correlated with dipole moment of analytes and was interpreted by an increasing nonradiactive recombination through the carriers’ diffusion (induced by analyte dipole moment) towards the surface traps [2]. In addition, it was demonstrated that PL intensity depends both on the nature of vapors and surface modification being sensitive to hydrophobic (as prepared PSi) and hydrophilic (oxidized PSi) properties [3]. In 1996, Bjorklund *et al.* [8] found a color change of PSi monolayers upon exposure to vapors of organic solvents as a result of a refractive index increase due to vapors adsorption inside the mesoporous structure. The following year, the same authors [9] reported a strong influence of water, acetone, and ethanol vapors on ellipsometric parameters of PSi monolayer. Snow *et al.* [10] observed a large wavelength shift in the reflectivity spectra of PSi Bragg reflectors (BR) upon exposure to chlorobenzene and acetone vapors. The spectral shift is caused by a refractive index change due to capillary condensation of vapors inside mesoporous silicon. Using modeling of reflectivity changes based on the Brugemman approximation, they were able to extract from experimental data the liquid volume fraction for acetone (0.29) and chlorobenzene (0.33).

Gas sensing with PSi is based on the adsorption of vapor molecules on the inner walls of silicon pores. There are two main mechanisms of analyte interaction with PSi: (i) microcapillary condensation and (ii) an actual surface sorption (physisorption) due to analyte interaction with PSi surface through Van der Waals forces. Microcapillary condensation, when vapors can spontaneously condense in a microporous and mesoporous material, can be described by the Kelvin equation [11]. At a given temperature, the smaller the pore size, the lower the partial pressure at which microcapillary condensation can occur. Usually, the microcapillary condensation is effective for relatively high vapor pressure, while, for lower concentrations, physisorbtion is the major mechanism responsible for changing the PSi optical properties. For example, in the study [12], it was suggested that microcapillary condensation is responsible for enhanced sensitivity to ethanol vapors for analyte concentration above about 10 ppm. Since the porous Si structure has a high surface area, a large number of analyte molecules per unit volume can be expected to adsorb, owing to physisorption. Nevertheless, as a rule, physisorption for as prepared PSi, even with a high interfacial area, cannot provide sufficient sensitivity for analyte concentrations in the mid/low ppb range to induce an observable spectral shift of resonance wavelengths or Fabri-Perot fringes (at least several nm) in the reflectance mode. Therefore, most of the early studies [2,3,8,9,10] employing reflectance spectroscopy utilized saturated vapors or high analyte concentrations (ppm range), resulting in microcapillary condensation in order to achieve a sizable increase of the refractive index. At this point, the importance of surface chemistry for higher sensitivity was realized and stimulated further investigation of PSi composites fabricated by various oxidation and carbonization methods (thermal, ozone, organic species, surfactants, chemical grafting, *etc.* [1]), polymer grafting, electrochemical growth and infiltration [13] (see next section). 

Another important advantage of the PSi functionalization is related to the improved specificity of sensory surface to target analytes. Indeed, changes in the reflectance spectra induced by different vapors with the similar refractive indices should be almost identical making their discrimination problematic. In contrast, the PSi surface covered by an organic layer can provide selective binding with analyte molecules, leading to enhanced specificity. In the case of PSi coverage by sensory polymers, the various analyte-polymer interactions can be quantitatively determined by the linear salvation energy relationship (LSER) [14]. Alternatively, improved selectivity can be attained utilizing multiparametric gas sensing in optical or optical-electrical domains. In this regard, Miloni and Pavesi [15] studied the spectral shift of resonance wavelengths of PSi microcavity (MC) in parallel with the intensity change of its photoluminescence upon exposure to organic vapors. They found that the spectral shift of the MC peak depends on the refractive index of analytes (liquids or saturated vapors) while the PL intensity correlates with their low-frequency dielectric constants. Thus, vapors with similar refractive indices (e.g., ethanol and pentane) but different dielectric constants can be readily discriminated. In fact, this study can be considered as a first report on multiparametric optical sensing with PSi structures (dual readouts). Later, Pavesi’s group reported more sophisticated triple-channel sensing (PL intensity, MC peak spectral shift, and resistance) using PSi MC for vapor detection and classification [16].

The PL quenching is more sensitive to analyte molecules than the shift of resonance wavelengths because of the different nature of these transduction mechanisms. The sizable spectral shift requires a change of the refractive index at relatively high analyte concentrations, while the PL quenching depends mostly on the energy transfer or the photoinduced charge transfer, which implies an amplification effect due to exciton migration within a single Si nanocrystal or through a nanocrystal network [17]. In particular, one analyte molecule (serving as a surface trap) is capable of capturing many excitons in Si nanocrystals resulting in efficient PL quenching. The confirmation of the above notion has been found by Content *et al.*, [18] studied PL of bare PSi monolayer, where detection limits for niroaromatic vapors (nitrobenzene, dinitrotolyene (DNT), and trinitrotoluene (TNT)) were determined as 500 ppb, 2 ppb, and 1 ppb respectively, which were significantly lower than typical VOC concentrations (saturated vapors or ppm range) utilized in the reflection spectroscopy experiments in the 1990s and in the beginning of the 2000s. However, despite the high sensitivity, photoluminescence of bare PSi could not attract further sufficient research interest because of its (i) low quantum yield; (ii) slow and inefficient quenching (several minutes are required to attain of ~5% signal reduction from initial PL intensity [18]) and (iii) non specific response. In order to provide specificity, the PSi should undergo chemical functionalizion, which usually quenches the PSi photoluminescence. Recently, we proposed a new concept based on the infiltration of the PSi multilayer structure with highly emissive fluorescent sensory polymers, allowing for the detection of several nitroexpolosive vapors with detection limit in ppt range [19,20,21] (see next section).

A systematical analysis of factors affecting the sensitivity of the PSi monolayer has been performed by Gao *et al.* [12]. It was demonstrated that low porosity layer is most sensitive upon ethanol exposure, resulting in the maximum change of optical thickness being consistent with the microcapillary mechanism. The layer thickness did not affect the sensitivity if the spectral shift of Fabry-Perot fringes was detected using broad band white source; however, it was strongly increased for layers with high thicknesses if the monochromatic light source (laser) was utilized. In parallel, the same authors investigated the effect of surface treatment on sensitivity, specificity, and stability of the PSi monolayer to vapors of ethanol, methyl ethyl ketone and hexane [22]. Obtained results demonstrated a value of surface modification for the enhanced selectivity of PSi optical gas sensors.

Another critical issue at this time was the development of a rational strategy for PSi surface modification to provide sufficient stability in long-term sensing applications. As prepared, PSi surface is hydrogen terminated and affected by environmental oxidation (by air or water), which causes undesirable changes in the material properties of PSi, such as zero-point drift in reflectance spectrum [1]. Therefore, the various methods of oxidations were developed to replace hydride species with different surface groups (thermal oxidation, ozone oxidation, oxidation by organic species, *etc.* [1]). A common method of creating a thin SiO_2_ layer is the thermal oxidation in air; however, silicon oxide is soluble in water, making such a structure not applicable for biosensing. This stimulated extensive research of alternative approaches through PSi carbonization (surface termination by Si-C bonds) [23,24,25] at the end of the 1990s/early 2000s that quickly yielded in excellent results, demonstrating a significant increase in the stability of the PSi surface. Afterwards, thermal carbonization and thermal hydrocarbonization has been successfully employed to improve the performance of gas sensors [26,27,28,29].

In the beginning of the 2000s, gas sensing with PSi MC in the reflectance mode has been extensively investigated by De Stefano *et al.* [30,31,32]. Using a time-resolved measurement of the red shift of MC resonance peak upon exposure to isopropanol saturated vapors, they concluded that microcapillary condensation occurs homogeneously in all layers (first Bragg reflector, MC, and second Bragg reflector) according to the consistency of experimental data (non-disturbed MC shape) with proposed model [32]. In another study, they demonstrated that the MC red shift is sensitive to hydrocarbon vapors and specific to their molecular weight [31]. In addition, time-resolved reflectivity has been applied to discriminate two vapors (acetone and methanol) or to identify their components in a binary gas mixture [33]. 

Letant and Sailor reported the detection of hydrofluoric acid (HF) gas using a simple interferometric technique by measuring the blue shift of Fabri-Perot fringes in reflectance spectrum [34]. They demonstrated that HF exposure reduces the optical thickness of the PSi monolayer due to conversion of surface Si-O and Si-F groups in gaseous SiF_4_. However, the detection limit was 30 ppm in 10 min and the spectral shift was irreversible. Using a similar transduction mechanism, Sohn *et al.* [35] showed that the simulant of orhanophosphorous chemical warfare agents (CWAa) can be detected with the PSi monolayer containing a copper (II) hydrolysis catalyst and surfactant.

## 3. Recent Progress in the Improvement of Sensitivity and Specificity of Gas Sensors with Multilayered Psi Structures

This section will focus on recent progress in PSi optical gas sensing based on multilayered structures, such as Brag reflectors, Rugate filters, microcavities, and especially designed stack of porous layers with different surface properties. The first part of the section will consider modified PSi surfaces, while the second part will describe gas sensing by PSi polymer composites, where sensory polymers plays a critical role in the improvement of sensor performance. 

### 3.1. Sensors with Modified PSi Surface

After the first reports demonstrating the importance of surface chemistry on the performance of PSi gas sensors [3,22], a significant effort has been made to investigate how surface modification could improve sensor sensitivity, specificity, and environmental stability. Ruminski *et al.* [28] carried out a systematic study of a spectral shift of resonance wavelength of Rugate filter upon exposure to VOCs as a function of six different surface modifications. The thermal oxidation and acetylene carbonization displayed an acceptable stability and different responses to isopropyl alcohol (IPA) and heptane vapors. The calibration curves (dependence of the spectral shift on analyte concentration) for IPA and heptane were sufficiently different and linear in the range of 50–800 ppm (except for the thermal oxidized sample for IPA), suggesting vapor classification and quantification. These two modified PSi and native PSi (as a reference) films were removed from the bulk substrate and attached to the distal end of bifurcated optical fiber similar to the procedure in previous work [36] where only native PSi was used. The fibers were tested upon exposure to 500 ppm of IPA in a carbon respiratory cartridge simulator. It was apparent that modified PSi samples exhibit improved stability during 10 min of IPA exposure (no blue shift) as compared with the native PSi (a blue shift of ~2 nm). 

The impact of surface carbonization on sensor stability and sensitivity has also been confirmed in a study [29]. Here, four types of PSi surface modification have been tested for amine vapors and ethanol. The results showed that acetylene carbonization/hydrocarbonization and functionalization by undecylenic acid (to graft carboxylic groups) provide excellent longterm stability upon exposure to methylamine and trietylamine vapors (no blue shift of MC peak was observed after the end of exposure) in contrast with native PSi functionalized by the undecylenic acid without carbonization (sizable blue shift), which was attributed to the oxidation of unprotected surface by amine vapors. Also, carbon modified surfaces demonstrated a higher sensitivity to amine and ethanol vapors. Another method of carbonization has been utilized in a recent study [37] where a PSi/carbon composite was prepared by thermolysis of an infiltrated precursor for the detection of flammable hydrocarbons (ethane, ethylene, and acetylene). Carbon modified Rugate filter exhibited increased sensitivity towards C_2_ hydrocarbons as compared with thermally oxidized PSi (references sample), and the detection limit was determined as 0.2% (v/v), which is more than ten times below the lower flammable limit. 

Long-term stability and reliability of PSi optical sensors are affected not only by natural oxidation, but also by temperature, humidity, fluctuation of light intensity, and noise. In order to minimize the influence of these factors on sensor reliability, the concept of the “reference channel” has been introduced in studies [38,39,40,41,42]. Usually, two PSi structures with different surface modification (lateral or stack) are fabricated with spectrally separated resonance peaks in the reflection spectrum in order to prepare two optical readouts (reference and signaling). Then, the difference in the spectral shift or intensity ratio between reference and signaling channels upon analytes exposure provides a compensation of undesirable factors and thereby improves analyte classification and quantification. In particular, one of the first “reference channel” approaches has been implemented for ammonia sensing [38], using double-peak Rugate filter infiltrated with a pH sensitive dye. Ammonia exposure results in a spectral shift of dye absorbance band overlapping with the first resonance peak and reduces its intensity (signaling channel), while the second resonance peak remains unchanged (reference channel). Thus, the ratio of the intensity of the two peaks is proportional to an ammonia concentration that is insensitive to large fluctuations in the light intensity. 

Another design of a reference channel concept was proposed in studies [39,40,41], where reference and signaling peaks correspond to two laterally separated spots with different surface modification on the same substrate. The reflectance spectra from both porous layers were recorded simultaneously. Such dual-channel PSi structures were employed for Cl_2_ gas sensing [39] (as etched PSi and PSi infiltrated with polystyrene); ethanol and toluene [40] (patterned with hydrophilic APTES and hydrophobic PFPS silane compounds); ammonia at different humidity levels [40] (oxidized PSi and PSi infiltrated with a pH sensitive dye). 

Ruminski *et al.* [42] investigated the humidity compensation sensor for VOCs using reference channel in a double-stack PSi structure comprised of two Rugate filters, one on top of the other. The top layer was modified to be hydrophobic (thermally hydrosilylated with 1-dodecene), while the bottom layer was hydrophilic (thermally hydrosilylated with undercylenic acid) and worked as a reference channel responsive to room humidity (RH). Apparently, the red shift of both resonance peaks is different in the presence of an analyte at certain RH levels. Then, the humidity interference with an analyte response can be compensated by the subtraction of a corrected spectral shift corresponding with the hydrophilic stack (reference channel) from the spectral shift corresponding with the hydrophobic stack (signaling channel). Authors were successful in the reliable detection of toluene, dymethyl methylphosphonate (stimulant of organophosphate nerve agents), heptane, and ethanol over a wide humidity range (25%–75% RH). 

The importance for the gas sensing of multilayer PSi architecture providing double peaks Rugate filters has been reported by Jalkanen *et al.* [43]. Rugate filters were fabricated by two different methods: stacking the top layer on the bottom one (similar to studies [42,44]) or by placing them in superposition using an etching current as a linear combination of two sinusoidal waves (similar to study [38]). Using ethanol vapors, it was demonstrated that the stacked Rugate filter reaches higher sensitivity compared to the superimposed filter. However, a superimposed filter can be prepared thinner than the stacked one, making for a faster recovery after analyte exposure. The triple Rugate superimposed filters were also utilized for ethanol vapor sensing [45], allowing for the quantification of ethanol concentration owing to linear dependencies of the red shift for each resonance peaks and ethanol dosing. 

In some cases, the enhanced selectivity of single PSi sensors in the identification of analyte vapors can be achieved without a reference channel and substantial surface modification; however, utilizing a sophisticated multilayer architecture, time-resolved reflectance spectroscopy, and thermal cycling [46,47]. Kelly *et al.* investigated the temporal response of a triple-peak Rugate filter in the reflectance mode monitoring the delay time between the spectral shift of peaks corresponding with the top and bottom layers [46].

**Figure 2:**
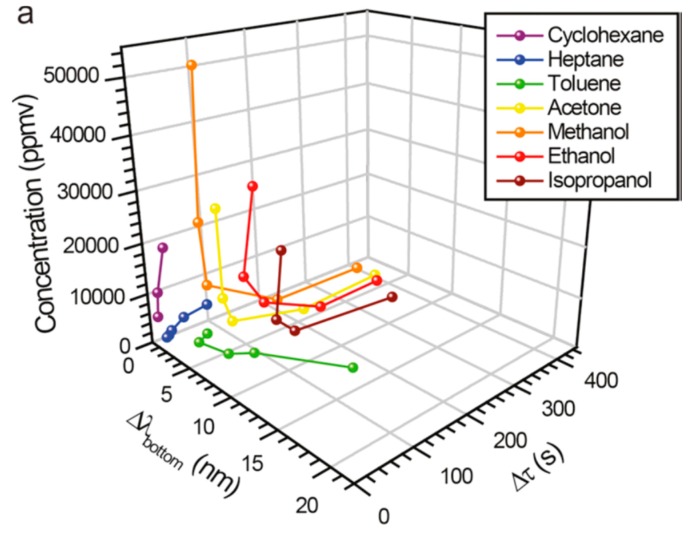
The 3D plot showing the wavelength shift of the photonic stop band of the bottom layer (Δλ bottom) and the retention time (Δτ) as a function of concentration for the seven analytes indicated. Reprinted with permission from Reference [46]. Copyright 2011 American Chemical Society.

The middle layer worked as a drift tube where the analyte diffusion affects the delay time depending on analyte concentration and polarity. This approach allowed for identification and quantification of seven different VOCs with concentration in ppm range (Figure 2). In the study [47], thermal cycling has been applied to a single-peak Rugate filter as an external factor, facilitating analyte identification (isopropanol, heptane, cyclohexane). The hysteresis of the spectral shift in the temperature cycle between 85 °C and 25 °C was specific to the analyte’s nature and attributed to diffusion and adsorption properties of each vapor. All of the above studies were mostly focused on the introduction of additional sensing parameter (e.g., reference channel, multiple-peak Rugate filter, time-resolved reflectance spectroscopy, thermal cycling) in the vapor detection scheme to improve sensor stability and analyte identification.

### 3.2. Sensors Based on PSi-Polymer Composites

Alternatively to PSi surface modification by small functional groups, PSi-polymer composite presents attractive functional material for gas sensing. Such composite structures can be prepared by various techniques, including direct polymer infiltration from solution, polymer grafting, *in situ* polymerization from monomers, *etc.* (see recent review [12] and references therein). Over the past decade, various PSi-polymer composites were designed and implemented mostly for biosensing [48,49,50], while surprisingly, only a few studies (discussed below) investigated the PSi-polymer structures as sensory material for optical gas detecting. Meanwhile, the integration of polymers with PSi scaffold opens new opportunities for developing highly sensitive optochemical devices possessing enhanced selectivity and stability. In particular, several advancements of polymer based composite over surface modification by small molecules/functional groups may include: (i) great chemical diversity of polymers allowing to design optical cross-reactive elements for sensor array tuned to different groups of target vapors [13]; (ii) strong enhancement of photoluminescent signal and device sensitivity [19,20,21], as compared with native PSi, utilizing various conjugated emissive polymers due to a high quantum yield and exiton energy migration through the polymer network , respectively [51]; (iii) the use of polymers as support for analyte sensitive dyes resulting in higher sensitivity and shorter response time in contrast to the dye itself [52]. In addition, in many cases, polymers can be infiltrated within a porous structure by simple coating/spin casting from solution, avoiding the multi-step surface modification generally required for tethering of functional groups to the inner walls of the nanoscale pores.

However, for vapor sensing applications the major challenge of polymer infiltration is the deep and uniform pores filling so that the polymer covers the pores walls as a thin film without pore clogging. The pores should be hollow and accessible for the interaction with analyte molecules from the outer atmosphere. Generally, such conditions can be satisfied through the polymer grafting [53], but this method requires end-functionalized polymers and a modified PSi surface by complimentary functional groups. This makes polymer grafting sufficiently complicated (similar to the PSi surface modification) and the applicability of this method is limited due to the relatively narrow set of appropriate functional groups. *In situ* polymerization inside the pores from small molecules (e.g., free-radical, photopolymerization, electrochemical, *etc.* [13]) is not very suitable for gas sensing, as polymerized material as a rule fills the entire pore volume. Thus, polymer infiltration from the solution remains the simplest and most appropriate technique for the design of gas sensors based on PSi-polymer composites.

One of the first reports on the improved performance of PSi gas sensors as a result of polymer infiltration was related to spectroscopic ellipsometery [54]. Authors demonstrated that the infiltration of PSi with amino-carboxyl-phenyl-thiophene based conjugated polymer and poly (acrylic acid) increased sensitivity up to 135% upon exposure to ethanol, methanol, and 2-propanol vapors. It was observed that enhanced sensitivity correlates with an optimal polymer concentration, while higher concentrations reduced the sensitivity, presumably due to the pores clogging. The rational choice of polymer concentration prior to the casting and proper solvent (to avoid aggregations), polymer molecular weight, gyration radii (should be less than the average pore diameter) and wettability of PSi by the polymer solution are the critical factors governing the uniform deposition of polymers as a thin film onto the pores’ walls. It is worth noting that polymer infiltration in nanoscale pores is a complex process when nano-confinement may affect the solvent evaporation and resulting properties of the deposited polymers. 

**Figure 3:**
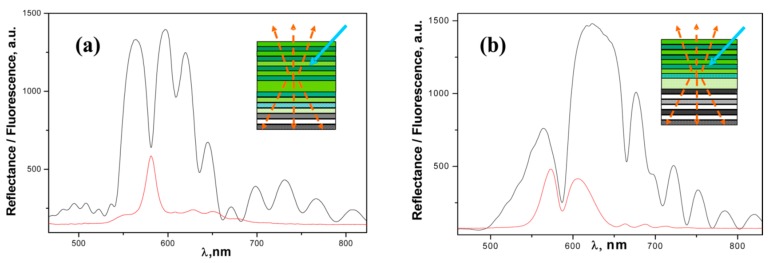
Reflectance (black) and fluorescence (red) of PSi MC infiltrated with Poly(2-methoxy-5-(2-ethylhexyloxy)-1,4-phenylenevinylene) (MEH-PPV) with a top (**a**) low porosity layer and (**b**) high porosity layer. Insets depict (**a**) deep and (**b**) shallow infiltration resulting in a resonance fluorescence peak and spectral “hole,” respectively (blue arrow—excitation light; yellow arrows—fluorescence; green color—infiltrated polymer). Reprinted with permission from Reference [21]. Copyright 2013 American Chemical Society.

In a series of our studies [19,20,21], PSi microcavities were infiltrated with conjugated emissive polymers in attempt to design and investigate new sensing composite materials for detection of nitroexplosives with low vapor pressure (e.g., TNT, cyclotrimethylene trinitraamine (RDX), pentaerythritol tetranitrate (PETN)), and interferant discrimination. It is known that some conjugated polymers exhibit a superior sensitivity to nitroaromatic vapors (parts per trillion range), including such explosives as TNT resulting in strong quenching of their emission [55,56,57]. The exciton energy migration along the polymer chain provides effective trapping and quenching of excitations generated by light, which is much greater than for isolated molecules (this phenomenon is also known as the effect of “amplifying polymers” [51]. Thus, the deep infiltration of such polymers inside PSi MC could combine merits of resonance PSi structure and highly sensitive sensory materials. It was found that deep polymer infiltration and formation of the thin film over pore walls is governed by the top layer of low porosity resulting in a fluorescence peak of the PSi microcavity [21]. In contrast, just switching the layers’ sequence so that the top layer was highly porous led to shallow infiltration and observation of the spectral “hole” in the broad band of polymer fluorescence (Figure 3). This phenomenon has been explained by a model taking into account the limit of capillary filling based on the balance of surface tension, viscosity, and vapor pressure forces and it was confirmed by secondary ion mass spectroscopy analyzing the profile of polymer infiltration along the MC depth [21].

**Figure 4:**
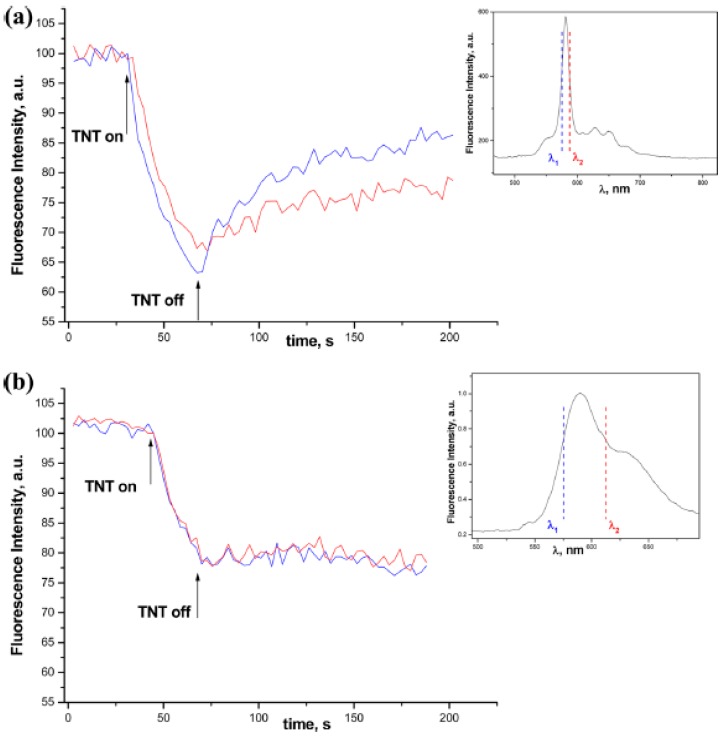
Time traces recorded at λ_1_ and λ_2_ wavelengths of the fluorescence intensity for (**a**) PSi MC infiltrated with MEH-PPV and (**b**) MEH-PPV film deposited on the flat Si substrate upon exposure to saturated TNT vapors (~7 ppb). Insets show λ_1_ and λ_2_ wavelengths on corresponding spectra with colors matching time traces. Reprinted with permission from Reference [21]. Copyright 2013 American Chemical Society.

Figure 4 shows enhanced sensitivity of the PSi MC-MEH-PPV composite to TNT vapors exceeding that of the conventional thin film of MEH-PPV deposited on flat Si. Such sensitivity gain can be attributed to the large interfacial area; extra thin, quasi-monomolecular polymer film deposited on the pore walls allowing for an effective analyte interaction with polymer; pore openings without clogging; and direct analyte diffusion inside porous structure owing to the capillary forces. The lower detection limit for TNT was estimated at 250−300 pg and 800−1000 pg for PSi composite and polymer flat film (thickness of 20 nm), respectively, using the “hot-wire” probe test. Another important feature of this sensor (associated with optical resonance in MC) is the spectral shift of the resonance peak upon vapors exposure. As a result, sensor specificity can be substantially improved by discriminating nitroaromatic explosives with low vapor pressure (spectral shift of ≤1 nm, e.g., TNT, Tetryl) from explosive interferants with medium and high vapor pressures (spectral shift of several nanometers, e.g., dinitrotoluene, nitrotoluene).

The influence of electrodeposited polypyrrol (PPy) on PSi photoluminescence quenching upon exposure to organic vapors (linear alcohols) has been investigated by Dian *et al.* [58]. For relatively low analyte concentration, the PL enhancement has been observed for PSi-PPy composites in contrast to PL quenching for native PSi. Authors claimed that such unusual behavior could be utilized for a design of the sensor array. However, lower signal intensity and a non-linear response with respect to native PSi makes such a design problematic. Additionally, the sensitivity of the composite sensor was lower than that of the native PSi, likely due to a high fill factor, which is typical for electrochemical polymerization *in situ*, as mentioned the above.

**Figure 5:**
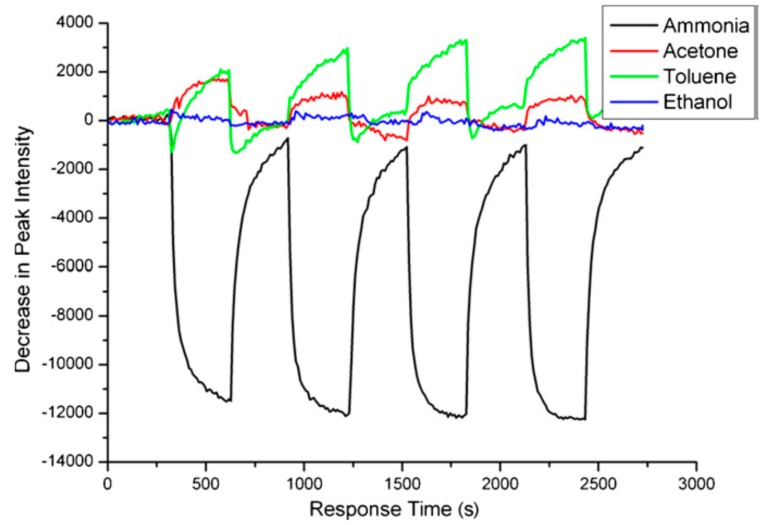
Optical response curve of the ammonia sensor to four kinds of gases. Reprinted with permission from Reference [52]. Copyright 2011 Elsevier B.V.

The polymer infiltration also improves the performance of gas sensors functioning in the reflectance mode. Shang *et al.* [52] demonstrated the importance of the polymer component for enhanced sensitivity, selectivity and stability. Similar to study [38], authors used bromothylmol blue dye absorption as an indicator of ammonia presence; however, the dye was loaded in supported cross-linked chitosan polymer infiltrated inside the PSi Rugate filter. This sensor, combining the value of chitosan polymer with the PSi resonance structure, exhibited better sensitivity and response time to ammonia as compared to the Rugate filter without polymer. Additional advancements of the polymer usage included good stability and enhanced specificity to ammonia, presumably owing to the hydrophilic properties of polymer layer preventing penetration of many organic vapors inside the PSi-polymer composite. Figure 5 shows the response of the sensor (reflectance intensity change at resonance wavelengths) upon ammonia and organic vapors exposure.

De Stefano *et al.* used PSi BR and BR with MC infiltrated with biocompatible polymer, amino-functionalized poly (ε-caprolactone) [59,60], for the detection of methanol, ethanol and isopropanol vapors [60]. The polymer was found to protect the silicon from alkaline solutions, allowing device to maintain the sensing capability after dipping in NaOH solution [60]. No comparison to the sensitivity of the reference samples (not affected by NaOH) has been presented. 

A strong impact of the polymer component on gas sensor performance was also demonstrated for photonic crystals other than PSi multilayered structures. Zhang *et al.* [61] reported an increase of the stopband red shift of the photonic crystal (SiO_2_ inverse opal) infiltrated with tetraphenylethene polymer as compared with the reference sample upon exposure to acetone and THF vapors. 

## 4. New Structures and Approaches

### 4.1. Nonperiodic, Superlattice, Surface Wave, and 2D Photonic Band Gap Structures 

Porous Si one-dimensional (1D) multilayered structures can be designed and fabricated by a nonperiodical manner, according to an algorithm determining the sequences and thickness of high and low porosity layers [62]. Most of these structures (e.g., Cantor and Thue-Morse) are also referred to as 1D fractal photonic crystals since their morphology is based on the self-similarity principle.

An example of the application of Thue-Morse multilayer (TMM) for gas sensing and comparison of its sensitivity with regular Brag reflector was reported by Moretti *et al.* [63]. A higher sensitivity of TMM was observed with respect to BR upon exposure to methanol, pentane, isopropanol, and isobuthanol, in terms of resonance wavelength shift and layer liquid fraction (filling factor). This fact was explained by the more effective capillary condensation for TMM structure due to the reduced amount of interfaces between high and low porosity layers as compared with BR. However, an alternative interpretation of these results can be associated with different pore morphology (sequences and thickness of high and low porosity layers) for BR and TMM, which critically determines the filling factor at capillary condensation [64]. As reported by Casanova *et al.* [64], utilizing the simplest PSi bilayers, the filling factors depends on relative vapor pressure (hexane) differently for “ink-bottle” (low porosity layer on top) and “funnel” (high porosity layer on top) geometry. Another example of the PSi fractal photonic crystal for sensing application is the Cantor multilayer investigated in the study [65] but without direct exposure to vapors. Here, standard PSi MC, as a reference and third generation of Cantor multilayer were modified by APTES and GTA (functional groups promoting biomolecule sorption inside the pores). Sensitivity towards surface modification for the Cantor structure (with low porosity layer for central part) was slightly enhanced with respect to reference MC. 

**Figure 6:**
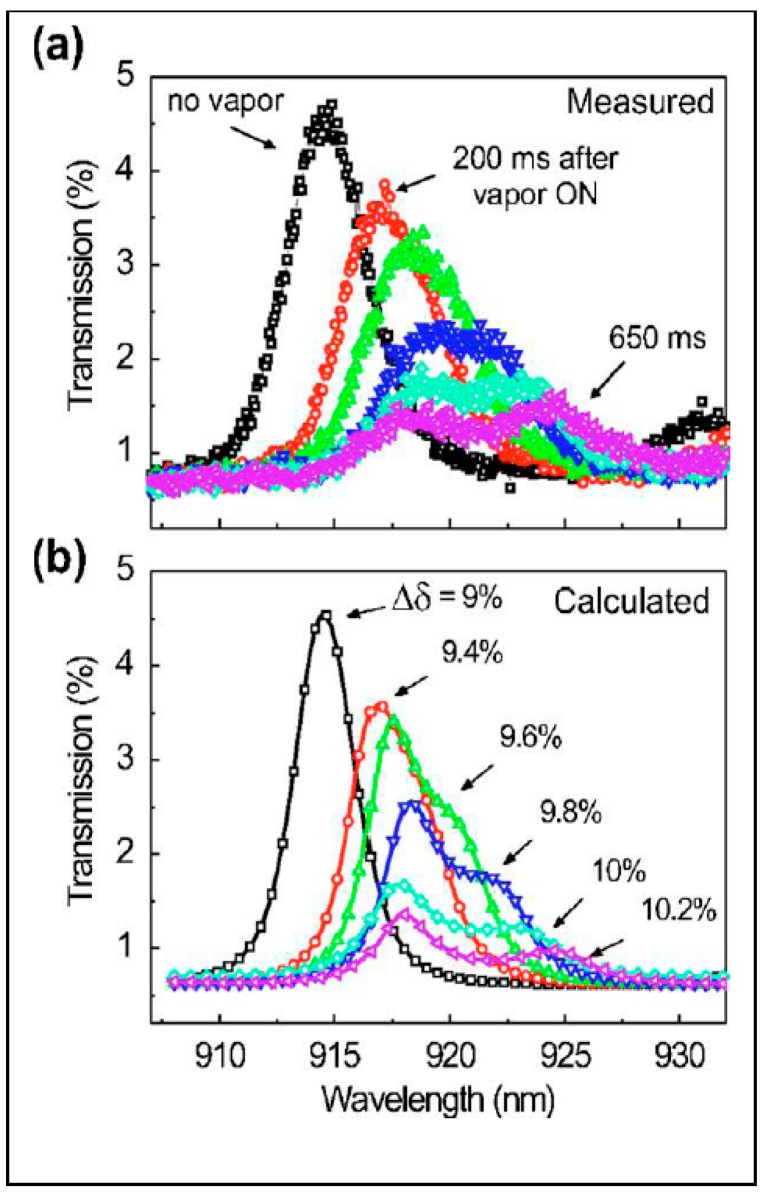
Vapor induced splitting of the Zener tunneling peak. (**a**) Experimental spectra of the sample exposed to ethanol vapors, (**b**) transfer-matrix calculations. The original double resonance peak splits into two separate modes when the built-in tilting is modified by the external “field”, mimicked by the refractive index gradient induced by the ethanol vapor flow. Reprinted with permission from Reference [66]. Copyright 2006 American Physical Society.

A sophisticated photonic band gap structure comprised of PSi superlattice for vapor detection has been investigated by Ghulinyan *et al.* [66]. An optical superlattice was designed as six supercells with two different microcavities, coupled through Bragg reflectors, with an optical path gradient, resulting in a tilted photonic band structure [66,67]. At critical band tilting, the light transmittance strongly increases due to Zener tunneling (similar to electrical Zener diode) when photonic minibands are coupled resonantly. Exposure by ethanol vapors modifies the refractive index gradient and disrupts the resonant Zener coupling conditions. As a result, the Zenner tunneling peak splits into two uncoupled peaks of reduced intensity. This phenomenon provides a fast gas sensing when a sizable change in transmittance occurs on a millisecond scale (Figure 6).

**Figure 7:**
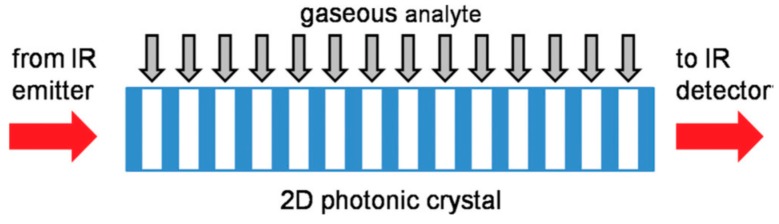
Schematic diagram of a typical 2D photonic crystal gas sensor. The light impinging from the left side is absorbed by the gas molecules inside the photonic crystal. Due to the reduced group velocity, the interaction path is effectively reduced. Reprinted with permission from Reference [71]. Copyright 2011 AIP Publishing, LLC.

The PSi two–dimensional (2D) photonic crystals were intensively investigated for the past decade mostly for biosensing in aqueous medium [62,68,69], whereas their applicability for gas detection was very limited, with the exception of the IR absorption spectroscopy [70,71]. However, gas sensing with 2D and 3D photonic crystals prepared from other materials has been sufficiently reported in literature (see recent review [72] and references therein). The fabrication of the PSi 2D photonic crystals requires combining precise photolithography with etching on a micron scale, which is a more complicated process than direct electrochemical etching employed for the fabrication of 1D periodical structures. Additionally, in 2D photonic crystals the light propagates normally to the pore axis, and its effective coupling with structures introduces an additional complexity (e.g., antireflection layer) in the photonic crystal design [71]. As the lattice parameter should commensurate with the light wavelength in visible NIR and mid-IR spectral range, only macrporous Si can be utilized in 2D photonic crystals. A new concept of gas detection based on spectroscopic absorption in mid-IR range and 2D PSi photonic crystal working as a gas cell has been proposed in the study [71]. The phenomenon of the low group velocity of the light passing through 2D photonic crystal (Figure 7) was utilized to increase the interaction of light with CO_2_ vapors. An enhancement of IR absorption by a factor of 2.6 to 3.5 was observed as compared with an empty gas cell, demonstrating the proof of concept. However, the performance of such an absorber is limited because of the requirement of precisely controlling the pore diameter according to numerical estimates (fluctuation should be less 0.5% for 1 mm device [71]).

The surface wave (SW) concept has been applied to gas sensing in order to gain enhanced sensitivity and a fast response time owing to the predominant interaction of analyte molecules with the surface of PSi 1D photonic crystal where the evanescent wave is propagated [73,74]. Initially in Otto [73] and later in Kretschman [74] configurations, authors demonstrated the spectral red shift of SW resonance peak for samples exposed to several VOCs at saturated vapor pressures. Time resolved kinetics, however, showed bi-exponential decay of peak position during vapors exposure that could be a drawback for the future development of the devices based on such method. In addition, the potential of SW approach for sensing application was investigated in the study, [75] using grafting amine groups to PSi surface and observed an increase of the coupling angle in the Otto configuration. 

The concept of the planar waveguide with Bragg grating has been exploited for VOCs (ethanol, IPA) sensing [76]. The resonance peak in transmission spectrum exhibited a fast and reversible red shift upon exposure to the pulse of organic vapors. The response and recovery time were determined as 0.3 s and 3 s respectively, demonstrating an excellent speed in device responsivity and reversibility. The Bragg grating structure in the core layer of the waveguide and the related transduction mechanism (spectral shift of the resonance peak) substantially improved the device performance as compared to early study [77], where only transmission losses were detected upon exposure to several VOCs.

### 4.2. Colorometric, Standoff, and Fiber Optic Methodologies 

**Figure 8:**
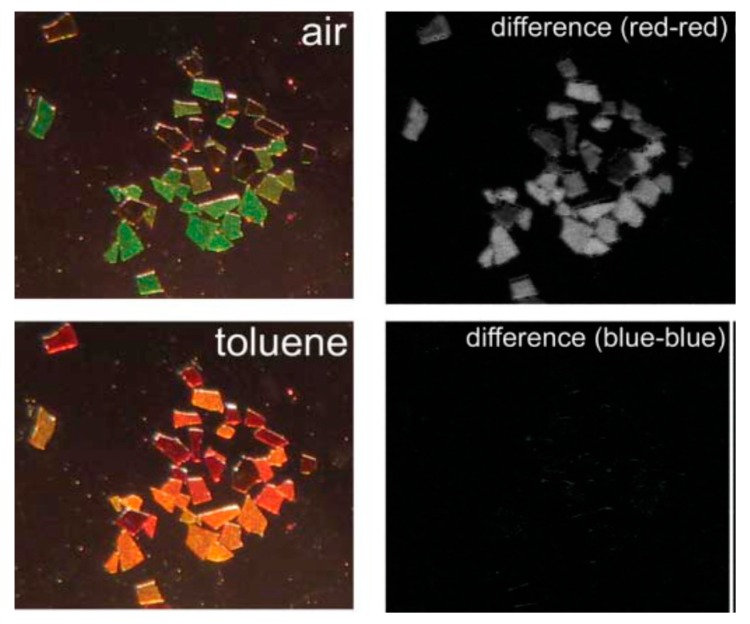
Sensing volatile organic compounds using ‘‘smart dust’’ photonic crystals. An array of microscopic porous Si photonic crystals exposed to toluene vapor is shown in these images. The **top left** image is the collection held in air. **Bottom left** is the sample after introduction of toluene vapor. The images on the right are difference maps, showing the difference between the red (**top right**) and blue (**bottom right**) channels. The size of these particles is of the order of 300 nm, and their surfaces are modified with dodecyl functionalities. Adapted with permission from Reference [79]. Copyright 2005 the Royal Society of Chemistry.

The concept of “smart dust” has been proposed by Sailor’s group in an attempt to extend the sensing capability to standoff vapor detection [78] and to simplify the response analysis through a color change that is visible to the naked eye [79]. “Smart dust” presents tiny particles (micron size) obtained by grinding or the sonication of the freestanding films of PSi photonic crystals (e.g., Rugate filters) removed from substrate. Depending on the etching current, the stop band of the Rugate filter can be positioned in Vis, NIR, and IR spectral range, providing a specific color encoding that is sensitive to the environmental changes. Because the stop band of the RF is relatively narrow, its spectral shift can affect the intensity of the reflected laser beam detected from a remote distance. Such standoff detection scheme was implemented in the study [78], where the intensity change of the reflected light of He/Ne laser from the “smart dust” photonic crystals (RF stop band was tuned to laser wavelength) was observed upon exposure to toluene, ethanol, and acetone vapors from a distance of 20 m using a photodiode mounted on the telescopic system. Another example of the use of “smart dust” for vapor sensing is the color change, attractive for a straightforward and rapid analysis by the naked eye [79]. Figure 8 demonstrates the photo images of the Rugate filter “smart dust” before and after exposure to toluene vapors, resulting in a drastic color change from green to red. 

**Figure 9:**
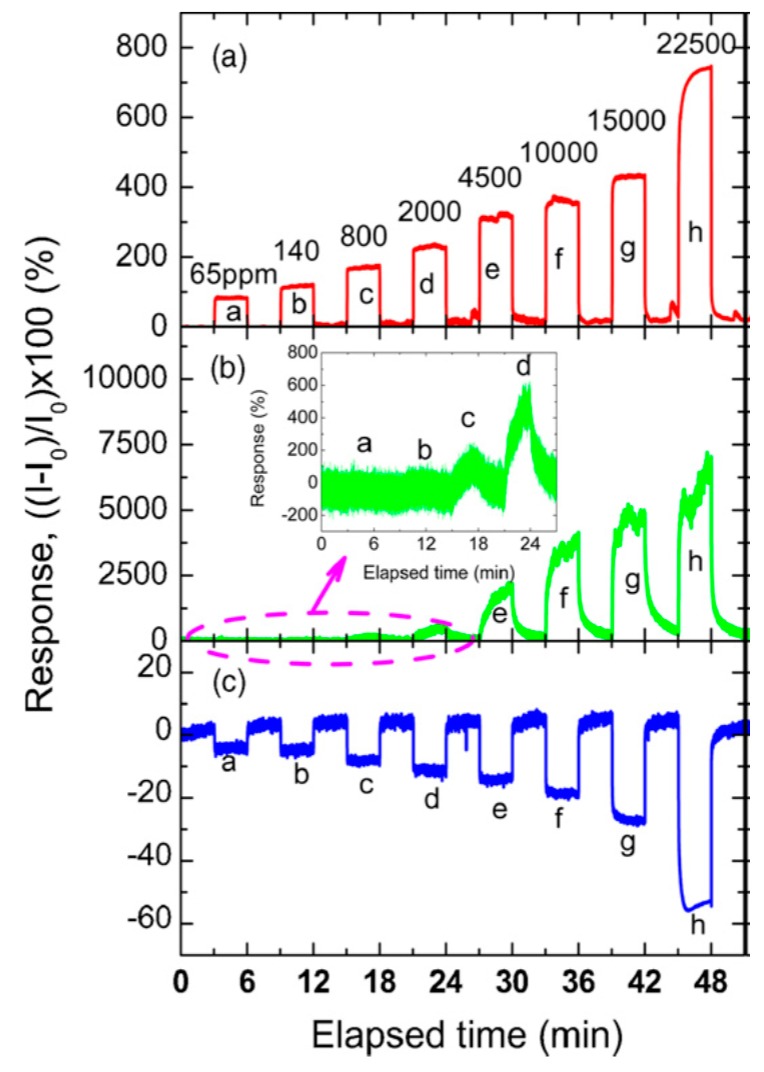
Comparison of the response signals measured by three different detection methods. This graph shows the response signals on the color-difference (**a**), current (**b**), and PL intensity (**c**). Reprinted with permission from Reference [80]. Copyright 2010 Elsevier B.V.

An impressive development of the colorometric technique, which allows for the quantification of the color change affected by the vapor presence, has been proposed by Park *et al.* [80]. The images of the PSi Bragg reflector during exposure to different doses of ethanol vapors were taken by a high-speed camera, followed by digital processing with a color-difference equation. Importantly that the color-difference method exhibited a higher sensitivity and a shorter response time than those of conventional luminescence and conductive transduction mechanisms (Figure 9).

PSi photonic structure can be removed from the substrate, cut down to micron size, and mounted on the distal end of the optical fiber for remote vapor monitoring in small and difficult to access volumes. In general, the fiber optic gas sensors have been widely explored over the past two decades due to several advancements over other sensing techniques in terms of miniaturization, flexibility, low consumable power, capability to build the distributed sensor network, and sensor array systems [81].

King *et al.* [36] investigated the possibility of detecting organic vapors breaking through the bed of activated carbon using the membrane of the PSi Rugate filter fixed on the cleaved end of the silica-core fiber. Such a sensor was able to detect IPA, cyclohexane, and thrichloroethylene vapors at the lowest concentrations of ~100 ppm being almost insensitive to the humidity level. For all tested organic vapors, the red shift of resonance peak occurred significantly faster than the reference response observed by the gas chromatography. Later, a similar approach was implemented in the study [28]; however, the Rugate filter membrane was chemically modified by thermal oxidation and carbonization as distinct from native PSi used in the previous study [36]. The fiber optic probe with carbonized PSi demonstrated a greater response to heptane than to IPA, whereas the probe with oxidized PSi displayed a greater response to IPA than to heptane. These results indicate that the combination of PSi surface modification with fiber optic probing could improve the selectivity of the fiber optic gas sensors. 

Another design of the fiber optic sensor based on the Rugate filter has been reported by Cho *et al.* [82]. Here, the RF membrane was mounted above the fiber distal end (on the surrounding round tube), allowing the simultaneous monitoring of the pressure difference (from both side of the membrane) owing to elasticity change and vapor (IPA) presence through the red shift of the resonance wavelength. 

One more example of the Rugate filter coupling with optical fiber (without removing the PSi membrane from the Si substrate) for gas sensing applications was demonstrated by Karacali *et al.* [83]. Instead of the typical fiber placement in front of the PSi surface, the authors utilized the Rugate filter structure coupled with fiber through a hole milled by REI at the back side of the Si substrate. The improved design was confirmed by a reduced mean deviation of the collected data and easy positioning of the probe. However, this performance was not compared with a free standing Rugate filter membrane glued on the distal end of the optical fiber as in previous studies [28,36]. 

## 5. Sensors Arrays and Multiparametric Gas Sensing

Despite great progress in PSi optical gas sensing over the past decade related to the improvement of the sensor specificity to target molecules, a major challenge still remains to accurately classify, discriminate, and quantify various gaseous analytes, especially those chemically close to each other, and analytes mixtures. Such problems are typical for any single (point) sensor based on a “lock-and-key” design possessing a high selectivity only to analyte of interests (or small group of analytes with similar properties). In order to improve the device recognition capability to a broader set of analytes and minimize false readings from possible interferants, the cross-reactive sensor arrays are as a rule utilized for various gas sensing applications [84,85]. In this regard, the previously discussed concepts of multiparametric [15,16,20,21,46] and “reference channel” [38,39,40,42,43,45,52] sensing can be considered from another point of view, notably as a prototype of a simple sensor array constructed from virtual independent sensory elements matching the number of readouts from a single, real sensor. For example, a multiparametric PSi single sensor providing independent readouts (electrical, luminescent and reflectance output signals) [16] can be presented as a simple sensor array comprised of three sensory elements. Additionally, two-parametric vapor sensing was reported in studies: (luminescence intensity/spectral shift) [15]; (capacitance/spectral shift) [86]; (current/spectral shift) [87]; (fluorescence intensity/spectral shift) [20,21]; (current/luminescence intensity) [88].

**Figure 10:**
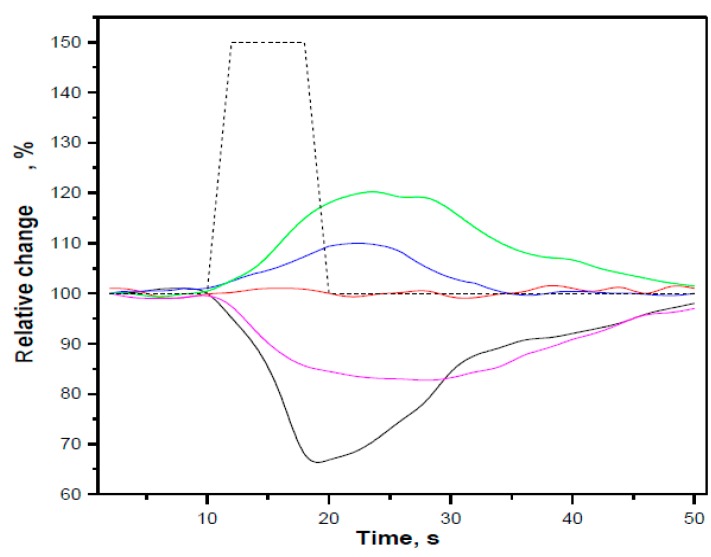
The temporal response of PSi MC infiltrated with MEH-PPV (Figure 2) on the pulse of TNT vapors (7 ppb, dotted line); black—the intensity of the fluorescent resonance peak; red—its spectral position; blue—resistance (R); green—capacitance (C); magenta—inductance (L). Three electrical signals recorded by the LCR meter (100 Hz) were synchronized with two optical signals (peak intensity and spectral position) through data acquisition system and LabView software [89].

Another example of a three-layer Rugater filter [46] resulting in discrimination of eight VOCs (Figure 2) demonstrates the ability of a single sensor with two independent readouts (spectral shift and retention time) to operate similarly to a two-elements sensor array. The “reference channel” approach [38,39,40,42,43,45,52] also suggests an additional readout as a second virtual element in the array, which improves the analyte classification and quantification. Figure 10 shows a five-parametric response of a single sensor, PSi microcavity infiltrated with MEH-PPV polymer, upon exposure of TNT saturated vapors. 

Furthermore, the multiparametric sensing is attractive for the array’s architecture because the output signals that are produced by different transduction mechanisms are mostly independent (orthogonal) and could significantly improve the cross-reactivity of the array’s elements. It is known that classification efficiency of any chemical sensor array critically depends on the chemical diversity of its recognition materials [14,84,85]. In many cases, insufficient chemical diversity is compensated by a large number of elements, making the array design complex and data processing unreliable due to multiple fluctuations determined by the number of elements. Multiparametric, orthogonal sensing could avoid such deficiency by reducing the number of sensory elements to several ones with the most prominent chemical diversity while maintaining the same number of output signals. 

Note that the above discussed approaches mimicking the sensor array architecture were employed for analyte identification without processing algorithms, except in several studies where the actual sensor arrays were implemented in conjunction with the correlation analysis [90] and pattern recognition (PR) [91]. 

Hutter *et al.* [90] reported ammonia detection using a sensor array composed of four PSi monolayeres infiltrated with pH sensitive dye (bromocresol purple) at different concentrations. The transduction mechanism was the reflectance change due to a spectral shift of the dye absorbance band reducing the reflectance intensity [38]. The correlation of the array output (vector of four signals) with an expected gradient-like response (owing to the gradient of the dye concentration) enabled the detection of ammonia minimizing the false positive from the humidity presence and unstable illumination.

**Figure 11:**
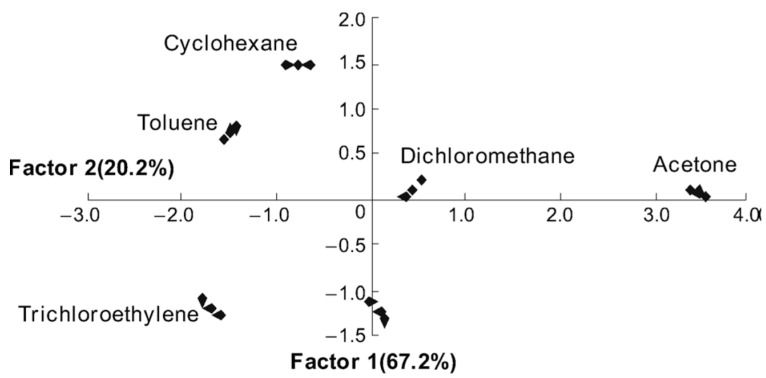
PCA-score plots obtained with the PSi/IL sensor arrays responding to six VOCs. The plot demonstrates that each VOC can be well-distinguished. Reprinted with permission from Reference [91]. Copyright 2011 Wiley-VCH.

The classical scheme of the optical PSi sensor array and PR algorithm has first been applied to the identification of the most common VOCs (typical environmental pollutants) in the study [91]. Here, the PSi Rugate filters were infiltrated with five different ionic liquids (IL) in order to provide maximum chemical diversity of the sensory elements. For this purpose, anionic parts of ILs were especially chosen to introduce the most types of analyte-receptor interaction according to LSER equation [14] (e.g., polarizability, hydrogen bond-acidity, *etc.*). The response of six elements in the array (five PSi/IL composites and one bare PSi Rugate filter) were measured to six VOCs (the peak spectral shift as a function of concentration), followed by the processing with the principal component analysis (PCA). The PCA is the PR algorithm reducing the dimensionality of the data set to canonical variances enabling graphical representation of the data [84,85]. Figure 11 shows an excellent clustering of different VOCs on a two-dimensional PCA plot demonstrating that different vapors can be completely recognized by PSi/IL sensor array.

## 6. Conclusions

Since the first reports of PSi optical gas sensors, significant progress in this field has been made in terms of sensitivity, specificity, response time, and environmental stability. The uniqueness of PSi structures for sensing application stems from several factors: the ability to fabricate various photonic structures with a large interfacial area and controlled porosity; easy surface chemical modification; versatility of PSi-polymer composites prepared by infiltration, grafting, and electrochemical growth; capability of multiparametric sensing, miniaturization, and integration with other functional Si modules in one photonic or optoelectronic circuit. Nevertheless, challenges still remain associated with accurate classification and quantification of various gaseous analytes (especially at low concentration and analyte mixtures) and sensor reliability in the real environment. Future research and development is envisioned as synergetic efforts involving multiparametric sensing, sensor array architecture with conjunction of PR algorithms, and miniaturization sensory system in one optoelectronic chip.
